# Cultural Context and Multimodal Knowledge Representation: Seeing the Forest for the Trees

**DOI:** 10.3389/fpsyg.2022.824932

**Published:** 2022-06-16

**Authors:** Melania Cabezas-García, Arianne Reimerink

**Affiliations:** Department of Translation and Interpreting, University of Granada, Granada, Spain

**Keywords:** context, culture, terminology, image, multimodality

## Abstract

Context, especially cultural context, has long been neglected in Terminology. Even though recent approaches have acknowledged the relevance of culture in specialized communication, the development of culture in Terminology is still marginal. Culture is also underrepresented in terminological resources, which may respond to the complexity of reflecting the cultural component in the description of terms and concepts. However, conceptualization is dynamic and changes from culture to culture and, for that reason, an in-depth study on how the nature of human perception and cultural cognition influences the representation of concept systems and terms in specialized knowledge contexts is needed. Furthermore, to facilitate knowledge acquisition, contextual and conceptual information should go together with multimodal information, as the combination of textual and visual material improves understanding. This study integrates different types of context (i.e., semantic relations, frames, and culture) to describe a methodology for the selection and representation of multimodal information for culturally bound concepts such as forest in terminological knowledge bases, based on the theoretical premises of Frame-Based Terminology. Different ideas of forest in European countries were analyzed and represented by means of culturally adapted images, which are best suited to disseminate knowledge and foreground the role of culture in specialized communication.

## Introduction

Context has been underexplored in Terminology, until the advent of new terminological currents (Gaudin, 1993; Cabré, 1999; Temmerman, 2000; L’Homme et al., 2003; L’Homme, 2004; Condamines, 2005; Diki-Kidiri, 2008 Faber, 2012), which acknowledged the need to study terms and concepts in communicative contexts. Furthermore, culture, although one of the main pragmatic aspects that can globally affect communication, has also been largely overseen in Terminology, as shown by the relatively low number of cultural studies in the field. A few exceptions can be found in Frame-Based Terminology (FBT; Faber and León Araúz, 2014; León Araúz and Faber, 2014), which foregrounds the role of context in knowledge acquisition, as well as in Culture-Bound Terminology (Diki-Kidiri, 2008), which emphasizes the fact that specialized communication can differ between speakers of different languages and cultures.

Culture is also underrepresented in terminological resources, which may respond to the complexity of reflecting the cultural component in the description of terms and concepts (Faber and Medina-Rull, 2017). Conceptualization is dynamic and changes from culture to culture and, for that reason, an in-depth study on how the nature of human perception and cultural cognition influences the representation of concept systems and terms in specialized knowledge contexts is needed (Faber and León Araúz, 2014, p. 135). Ideally, terminological resources should reflect the contextual dynamism of terms and concepts. Otherwise, there is the risk of prioritizing one culture over another and resulting impoverishment of underrepresented cultures.

Furthermore, contextual and conceptual information should be complemented with multimodal information for the sake of knowledge acquisition, as the combination of textual and visual material improves understanding (Cook, 2006). In our opinion, the selection criteria for multimodal information, such as images, to illustrate concepts and enhance knowledge acquisition should also be based on the cultural context since the prototypicality of mental images is culture-bound (Giora, 1997; Barsalou, 2003; Losin et al., 2010).

Different cultures and languages have shown to segment and label the large-scale environment and its features according to very different semantic principles and seemingly basic concepts like mountain, valley, and river cannot in fact be straightforwardly translated across languages (Burenhult et al., 2017). Forest is one of those culturally bound entities, which is very important to human beings because of how it influences their way of life from basic possibilities for shelter and construction to being a large part of their economic activity (Frick et al., 2018). Van Putten et al. (2020), for example, found that the concept forest is particularly variable across languages.

To the best of our knowledge, there is no research on the selection of prototypical images that illustrate different cultures and perceptions in terminological resources. In this study, we propose a methodology for the selection and representation of multimodal information for culturally bound concepts such as forest in terminological knowledge bases (TKBs), based on the theoretical premises of Frame-Based Terminology (Faber et al., 2006; Faber, 2012). FBT is a cognitive approach to domain-specific language, which directly links specialized knowledge representation to cognitive linguistics and cognitive semantics. In FBT, knowledge acquisition begins at the term-level, progresses to the phrase level, and finally results in the codification of an entire knowledge frame. For the purposes of this study, we started from the different ideas of forest in European countries, as described in the EEA Technical Report No 9/2006, *European forest types: Categories and types for sustainable forest management reporting and policy* (European Environment Agency, 2007). We developed a definitional template for forest and included cultural dimensions in the template converting it into a semplate (Burenhult and Levinson, 2008, p. 144), which facilitated the selection of culturally adapted images.

The rest of this paper is organized as follows. Section “Context and Culture” summarizes some of the studies on context and culture. Section “Multimodal Knowledge Representation in Frame-Based Terminology” explains multimodal knowledge selection and representation in FBT. The method and results of the study are presented in Sections “Materials and Methods” and “Results,” respectively. Finally, Section “Conclusion” summarizes our conclusions as well as our plans for future research.

## Context and Culture

Context is of paramount importance in communication. Some theories even consider that meaning is totally context-dependent (Barsalou, 1993, 1999; Coulson, 2000; Croft, 2000; Evans, 2006; Evans and Green, 2006). Given its relevance, multiple approaches to context have emerged. Coseriu (1967, p. 313) describes context as “a set of non-linguistic circumstances that are directly perceived or known to the speaker.” Escandell (2013) conceives it as “everything that, physically or culturally, surrounds the communicative act.” Kecskes (2014, p. 128) defines context as “any factor that affects the actual interpretation of signs and expressions.” We agree with Kecskes (2014) and share this idea of context, which can be further specified, for example, in terms of culture, the communicative setting, and linguistic cotext.

Concepts do not exist in isolation, but rather as part of a conceptual network, which facilitates concept understanding (Van Putten et al., 2020) and determines the dynamics of concepts and terms (León Araúz and Faber, 2014). Terms and concepts can vary in different disciplines, cultures, and communicative situations, among other contextual factors. Along these lines, different typologies of context have been proposed, which often overlap. For instance, Dik (1989) describes three types of context: (i) general, which includes world knowledge; (ii) situational, which encompasses the knowledge derived from the communicative interaction; and (iii) contextual, which consists of the linguistic expressions in the discourse. Similarly, Evans and Green (2006, p. 221) distinguish the following types of context: (i) encyclopedic; (ii) sentential; (iii) prosodic (intonation pattern); (iv) situational; and (v) interpersonal. Fetzer (2017) highlights the existence of cognitive context and distinguishes between: (i) linguistic context or cotext (e.g., clause, sentence, utterance, and text); (ii) social and sociocultural context (i.e., context of a communicative exchange, e.g., participants, time, and location); and (iii) cognitive context (i.e., mental representations and contextual assumptions). Reyes (2019) also argues for the existence of linguistic, situational, and sociocultural context. As pointed out by Kecskes (2014), context does not affect communication within the same culture in the same way as between different cultures. Along these lines, systemic functional linguistics (Halliday, 1989) studies the relationship between languages and social settings and highlights the role of cultural contexts in communication when distinguishing between contexts of situation and contexts of culture. Therefore, among the contextual factors that influence communication, the linguistic context, communicative situation, prior world knowledge, and culture should be highlighted.

Different ideas of context can be found in specialized communication. The distinction between local and global contexts has been frequent. Akman and Bazzanella (2003) describe local contexts as specific settings where participants interact, whereas global contexts include the members of a community and their circumstances. Mihalcea (2007) uses the same distinction to refer to a smaller or larger context span. Alternatively, Dash (2008) describes a continuum of four types of contexts: (i) local (the immediate context of a word); (ii) sentential (syntactic-based context); (iii) topical (domain-based context); and (iv) global (extralinguistic context).

According to Faber and León Araúz (2016), local contexts are usually limited to the words within the term itself, to a small number of words in the immediate vicinity of a term, or to words connected by dependencies to the term. Alternatively, global contexts can have a much larger scope and refer to a whole document, a communicative situation, a subject domain, or an entire language-culture. Both local and global contexts can be syntactic, semantic, or pragmatic.

On the one hand, local syntactic contexts are those that reflect the recurrent structural patterns of the term, such as multiword terms. For instance, the local syntactic context of *forest* includes multiword terms such as *beech forest*, *broadleaved deciduous forest*, and *forest management*. Rather than focusing on the formation of multiword terms from a given term, local semantic contexts either consist of the semantic relations between the constituents of a multiword term (e.g., in *forest management* > management *affects* forest) or to semantic relations between different concepts in the text. Local pragmatic contexts refer to parameters of terminological variation and culturemes. For example, depending on the level of specialization of the communication, the term variants *Fagus sylvatica forest* and *beech forest* can be used. *Beech forest* is preferred in non-expert communication, whereas *Fagus sylvatica forest* is more specialized. Term variation can also respond to cultural differences, as in *dry lake* and *sabkha*, the latter being a culture-specific term used as a term variant, which does not allude to exactly the same concept as *dry lake* but refers to the closer entity in the target culture (e.g., in North Africa; Faber and León Araúz, 2016).

On the other hand, global syntactic contexts are the different types of grammatical cohesion that tie the text together, such as discourse markers. Global semantic contexts are reflected in the lexical cohesion of texts. This is evident when a text or discourse is based on concepts of the same domain (e.g., a text about forests includes concepts such as tree, leaf, plant, and climate), which are linked by means of anaphora, repetition, and hypernymy. Finally, global pragmatic contexts can include variation stemming from culture, language users, and language use.

Culture, understood generally as the ways of life, customs, knowledge and degree of artistic, scientific, and industrial development of a group of people, is one of the main pragmatic aspects that can globally affect communication. Cognition is culture-dependent since our perception cannot be isolated from our environment and our previous experiences. These influence our categorization and neural plasticity (Losin et al., 2010). Along these lines, specialized conceptual categories traditionally considered to be universal have been found to be constrained by cultural perceptions. For example, ice-produced erosion will be more prototypical in language-cultures in Arctic regions (Faber and León Araúz, 2014).

Similarly, we believe that the selection of multimodal information, such as images, to illustrate concepts should also be based on the cultural context since the prototypicality of mental images is also culture-bound (Giora, 1997; Barsalou, 2003; Losin et al., 2010). The prototypical image of a landscape for inhabitants of Finland differs from that of the inhabitants of Saudi Arabia. A common landscape for Finns may be a polar forest, while a typical landscape for Saudis may be a desert. Along these lines, Frick et al. (2018) monitor Swiss residents’ perceptions on forests. They asked people questions regarding: (a) knowledge about forests, (b) preferences for certain forest types and features, and (c) the attitudes people hold regarding the importance of different functions of forests. Even though they did not focus on obtaining a prototypical image of forests, they found that respondents were well informed about forest issues, especially about recreation, animals, and protection from natural hazards. Nevertheless, functions such as wood production, air quality, and biodiversity were rated as even more important than recreational functions. Not surprisingly, these views can change in different cultures.

This is supported by the pragmatic notion of *salience*, which alludes to information that is central to our consciousness. The most salient information for one person is that which is most related or known to him or her. Evidently, what is salient for one person might not be so for another. Salience is directly related to prototype theory (Rosch, 1978), which claims that categories are organized by reference to prototypes, which are mental representations of the most characteristic member of a category (i.e., the most salient). Geeraerts (2000) investigates the notion of salience in Lexical Semantics and claims that some senses are more salient than others because they are more readily chosen when using that category. Therefore, Geeraerts (2000, p. 80) describes salience as the most probable out of all possible interpretations of a lexical unit. Salience also depends on intentionality. When communicating about a particular subject, concepts related to that subject are the most salient ones (Kecskes, 2014). Therefore, salience guides language production (Myachykov, 2007) and language processing (Giora, 1997; Geeraerts, 2000). Grounded cognition of Barsalou (2003) applies here because our perceptions are driven by our personal experience and are stored in the form of mental representations.

Multiple term variants can also emerge as a result of conceptualization in different cultures. In those cases, the one that is most appropriate to the receiver, his/her knowledge and culture, communicative situation, and text genre must be chosen. Additionally, cultural differences can lead to terminological gaps. The term *swamp* has no equivalent in Russian because in this culture there are few forested wetlands that are not simply a variety of peatlands (Mitsch and Gosselink, 2011). Likewise, Faber and Medina-Rull (2017) describe terms for local winds that occur in one cultural context but not in another. Similarly, in Duna, a Papuan language spoken in New Guinea, the term *rowa* includes the meanings of *tree*, *firewood*, and *fire* (Schapper et al., 2016), and also extends to instruments of fire, like *matches* and *lighter* (Burenhult et al., 2017). This conceptual association is also found in other indigenous languages in the Australia-Pacific region (Burenhult et al., 2017). Such conceptual asymmetries may be an obstacle to understanding when receivers belong to a different culture. Thus, context-modulated information should be available for potential activation when the user of a terminological resource wishes to acquire knowledge about it (Faber and León Araúz, 2014, p. 140).

Cultural differences have been mainly studied in the domains of motion (Talmy, 2000), the body (Enfield et al., 2006), and the senses (Majid and Levinson, 2011). In environmental science, the concept of landscape has received particular attention (Burenhult and Levinson, 2008; Villette and Purves, 2019; Van Putten et al., 2020), up to the point that there is a discipline called ethnophysiography, which investigates the influence of landscapes on language use and its variation between cultures. Evidence gathered in ethnographic and linguistic studies contradicts landscape universality and points to an astonishing diversity in ways of conceptualizing and referring to landscape (Burenhult and Levinson, 2008). In particular, Van Putten et al. (2020) found that European languages conceptualize landscapes differently. For example, in France, landscapes often involve the presence of water, which is not always the case in other European countries.

Forests, which belong to the landscape category, have been studied in Burenhult (2009), Chazdon et al. (2016), Burenhult et al. (2017), Côte et al. (2018), Comber and Kuhn (2018), and Frick et al. (2018), *inter alia*. The notion of forest is seemingly straightforward and similar sets of parameters are often used in their definitions, such as “tree height,” “canopy cover,” and “land size” (Burenhult et al., 2017; see Section “From Template to Semplate”). However, there are culture-based gaps and differences between the concepts evoked by forest, as suggested in Van Putten et al. (2020) regarding the different conceptualizations of landscapes in Europe. Such cultural mismatches are perceptible in definitions (Comber and Kuhn, 2018) or, as suggested by Faber and Medina-Rull (2017), in definitional templates. These templates include the semantic relations encoded by the concept (Faber, 2002), which are filled with different values depending on the culture in question. The selection of a particular value to fill the template does not respond to particular preferences, but to cultural salience (Côte et al., 2018). Along these lines, Côte et al. (2018, p. 254) suggest that culture-bound concepts should be described by means of different, culture-adapted definitions. Chazdon et al. (2016) and Comber and Kuhn (2018) support this idea and claim that no single operational forest definition can, or should, embody all of the dimensions activated by the concept forest throughout the world. An example can be found in the *Compilation of Forestry Terms and Definitions* from the European Forest Institute Internal Report No. 62002 (Schuck et al., 2002), which includes different forest definitions for every European country. If, however, a particular view or definition of forest is prioritized over another, marginalization of local community voices and local landscape conceptualizations is a consequence (Comber and Kuhn, 2018).

Burenhult et al. (2017, p. 456) explain the case of Lowland Chontal, an endangered indigenous language of Oaxaca (Mexico). In this language, there is the term *muña*, which apart from incorporating meanings similar to English *forest* or *jungle*, also alludes to bush, underbrush, overgrown wilderness, or any type of weeds or garbage. Besides, the term invokes a wider meaning of a disorderly environment, one that is not kept in check by humans. An entity referred to as *muña* does not have to contain good-sized vegetation in the form of trees or in fact any vegetation at all, as occurs in most languages. Similarly, although the people of Jahai in the Malay Peninsula live in forests, they have no concept of forest (Burenhult, 2009). Instead, forests are regarded as their home and the nearest terms they have for forest-like concepts refer to leaves and trees, canopy floor, covered area, and exposed area (Comber and Kuhn, 2018).

These studies are undoubtedly useful for understanding forest conceptualization. However, to the best of our knowledge, there is no research on the selection of prototypical images that illustrate different cultures and perceptions in terminological resources. This may be due to several reasons. First, context has been underexplored in Terminology, until the emergence of new terminological theories (Gaudin, 1993; Cabré, 1999; Temmerman, 2000; L’Homme et al., 2003; L’Homme, 2004; Condamines, 2005; Diki-Kidiri, 2008; Faber, 2012), which claimed that terms and concepts should be studied in communicative contexts. Second, the complexity of considering culture in terminological work has probably affected the number of cultural studies in Terminology. Exceptions include FBT (Faber and León Araúz, 2014; León Araúz and Faber, 2014), which highlights the relevance of context in knowledge acquisition, as well as Culture-Bound Terminology (Diki-Kidiri, 2008), which claims that specialized communication can vary in different languages and cultures.

This cultural facet is also underrepresented in terminological resources, which may also respond to the complexity of reflecting the cultural component in the description of terms and concepts (Faber and Medina-Rull, 2017). Ideally, the contextual dynamism of terms and concepts should be described in terminological resources. Otherwise, one culture would be prioritized over another, which would impoverish underrepresented cultures. Moreover, to enhance knowledge acquisition, multimodal data should be an added value to contextual and conceptual information since the combination of textual and visual material facilitates understanding (Cook, 2006).

## Multimodal Knowledge Representation in Frame-Based Terminology

Much has been written regarding the importance of combining visual and textual information to enhance knowledge acquisition (Paivio, 1971, 1986). FBT (Faber et al., 2006; Faber, 2012) has always stood for multimodal knowledge representation in terminological resources to help users acquire the specialized knowledge they need as thoroughly and quickly as possible. However, the selection of visual content cannot be random or based on intuition and thus, the combination of images and text still needs further analysis (Prieto Velasco and Faber, 2012; Reimerink et al., 2016). An in-depth analysis of the features of images provides the means to develop selection criteria for specific representation purposes (León Araúz et al., 2019). The combination of conceptual content, image type based on morphological characteristics, and functional criteria can be used to enhance the selection and annotation of images that explicitly focus on the conceptual propositions that best define concepts in a TKB.

In FBT, a set of criteria was developed for the selection of images based on the interaction between concept type, image type, and visual knowledge patterns (VKPs), which we define as the images’ morphological features, such as the use of colors, arrows, and labels (León Araúz and Reimerink, 2016; Reimerink et al., 2016).

Two functional criteria are applied, referential similarity and dynamism, to analyze VKPs in images. Referential similarity refers to the degree to which an image resembles its referent in the real world. This similarity is measured on a continuum ranging from non-similar to totally identical. It goes without saying that a two-dimensional image can never be totally identical to its referent, but a color photograph would have a high degree of referential similarity. Dynamism can also be measured on a continuum ranging from totally static to very dynamic. Dynamicity in images can be enhanced by the use of VKPs such as arrows, for example, that connect one phase of a process to another, or even by using several static photographs together that show the result of the different phases of a process. The results of our research showed which VKPs and which degrees of referential similarity and dynamism are most characteristic of different types of images and how they are related to the conceptual propositions represented in each type (Reimerink et al., 2016).

For instance, processes are generally described by the meronymic relations *phase_of* and *takes_place_in* because processes are composed of different stages and occur within a certain context. This is in direct contrast to physical objects, whose description is dominated by the relations *has_location* and *part_of*. Not surprisingly, processes are generally portrayed by flow charts that represent more than one relation and physical objects can often be clearly portrayed by photographs and drawings.

It has also become clear that VKPs, such as arrows, labels, and color-coding, are polysemic since the same pattern can be used for different purposes in the same way that textual knowledge patterns can also convey different conceptual relations (León Araúz et al., 2009). Accordingly, the conceptual knowledge underlying VKPs can only be interpreted in the context of each image. Nevertheless, a certain combination of patterns, constrained by image and concept type, makes images more or less suitable for the representation of certain types of conceptual knowledge. An arrow, for example, can be used to connect a term to its representation in the image; thus, this VKP does not necessarily transmit dynamism. However, when arrows appear in an image representing a process, they generally convey dynamism and go in the direction of the different phases of the process. The same is true for colors. In images with a high level of referential similarity, the colors in the image are the same or similar to those of the real world entity. In many cases, however, the function of the colors is not to realistically represent the concepts or its natural surroundings, but rather to differentiate closely related concepts in time or space.

**Figure 1:**
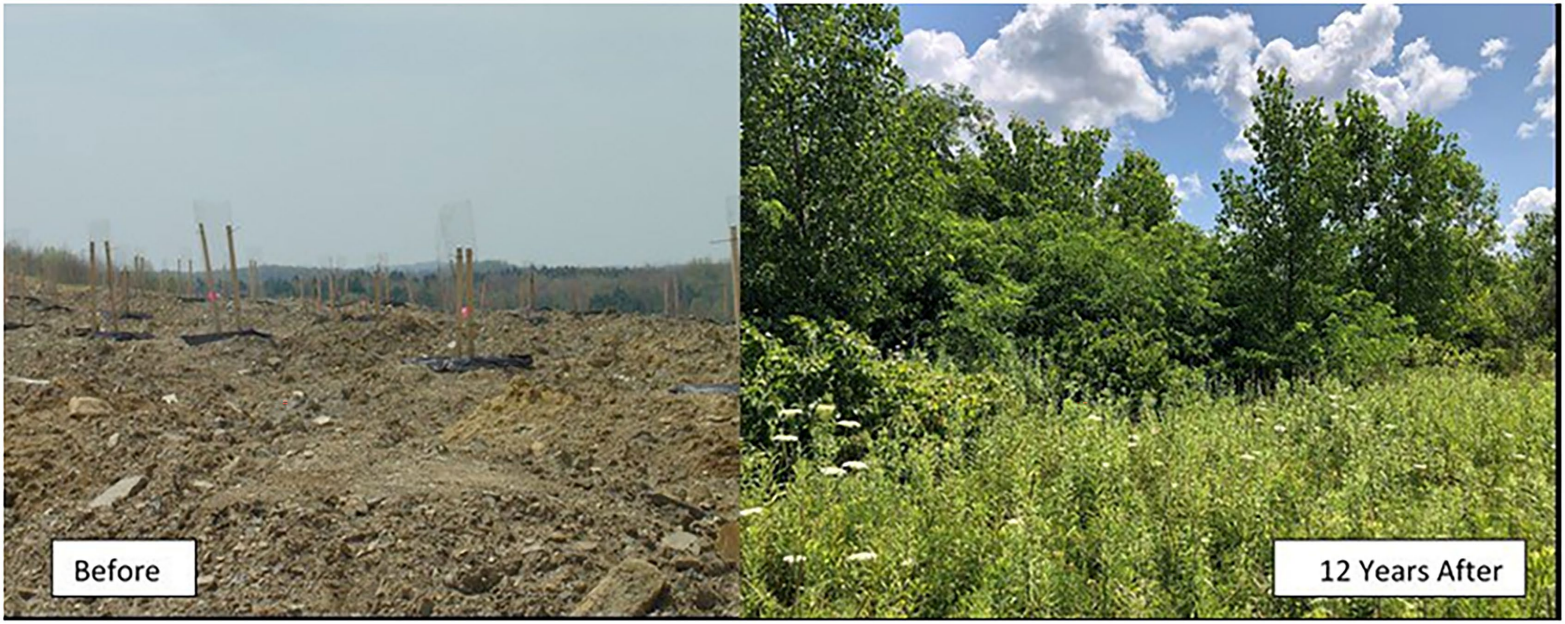
Image for reforestation *has_result* forest. *Source*: The American Chestnut Foundation. Reproduced with permission from The American Chestnut Foundation (TACF), available at https://acf.org/science-strategies/restoration/.

For example, reforestation is the process of replanting an area with trees. To represent this process, photographs of people planting trees can be useful. Such images show the start of the process and highlight the facet of human intervention (reforestation *effected_by* human). Furthermore, as trees take some time to grow, the evolution of the reforestation process can be shown over time. To give a more complete representation of the process, Figure 1 could also be used, which shows the before and after of the process.

**Figure 2:**
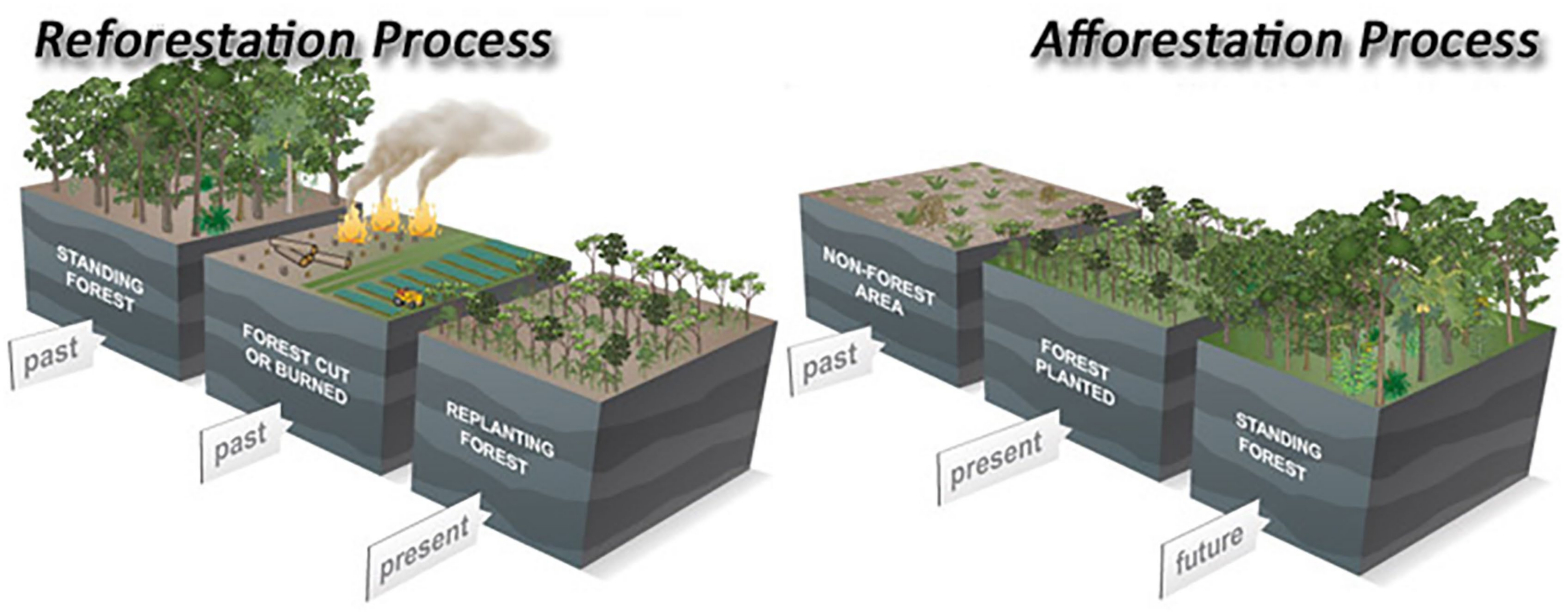
Image to differentiate reforestation from afforestation. *Source*: Geoengineering Inquiries. Reproduced with permission from Ken Caldeira, available at https://www.annualreviews.org/doi/abs/10.1146/annurev-earth-042711-105548.

Often complex processes are hard to represent with photographs alone, although in the case of reforestation, combinations of photographs work well as the concept refers to a process that makes physical objects evolve. A combination of images can also be helpful to differentiate two closely related concepts as in Figure 2.

In Figure 2, the combined drawings are more abstract (lower referential similarity) than the previous images, but a higher level of abstraction allows for the representation of more complex information. Colors are used not only to provide referential similarity as in the trees, the fires, and crops, but also to clearly distinguish between the three different time blocks (past, present, and future) and the two different concepts, reforestation and afforestation. The labels with text distinguish between both processes and add further explanations.

To explain how images are selected for TKB inclusion according to FBT, we will use the example of the concept tree. In EcoLexicon,^1^ a multimodal and multilingual TKB on the environment elaborated according to the theoretical premises of FBT, great effort is taken to provide consistent knowledge representation in all the modules of the TKB. EcoLexicon users include language specialists (e.g., terminologists, translators, and linguists), environment experts, and the general public. It is designed for anyone who needs to access to environmental concepts to understand, write, or translate specialized or semi-specialized texts. EcoLexicon’s interface has different modules of conceptual, linguistic, and graphical information, which can be chosen depending on user interests. FBT research is currently focused on the design of a cultural module, which will involve term annotation based on usage preferences and the selection of multimodal information adapted to the cultural context of the user, as described in this research.

The definitions in EcoLexicon are based on templates that define category membership and describe the basic conceptual propositions in which the concept participates. In this way, definitions have a uniform structure that directly refers to and evokes the underlying conceptual structure of the domain.

When applying a template to a concept, it may only inherit the relation with the defined concept in the template or activate a more specific concept than the one in the template. An example would be the template^2^ for tree (Table 1), which is applied to the definition of oak tree (Table 2) and holm oak (Table 3), both members of this category and each other’s direct hyponyms.

**Table 1:** Tree definitional template.

TREE	
Large, tall, woody, and perennial plant with a single, unbranched, erect, and self-supporting stem holding an elevated and distinct crown of branches, which can grow greater than 10 feet in height and greater than 3 inches in diameter.	
*type_of*	plant
*has_part*	root
	trunk
	branch
	twig
	leave
	crown
*has_attribute*	height
*has_location*	earth’s surface

**Table 2:** Oak tree definitional template after the application of the tree definitional template.

OAK TREE	
Any tree of the genus *Quercus* of the beech family that produces acorns and typically has lobed deciduous leaves. Oaks are dominant in many north temperate forests.	
*type_of*	tree
*has_part*	lobed deciduous leaf
*has_location*	north temperate forest
*produces*	acorn

**Table 3:** holm oak definitional template.

HOLM OAK	
Evergreen Mediterranean oak tree with evergreen ovate-lanceolate-shaped leaves.	
*type_of*	oak
*has_part*	evergreen ovate-lanceolate-shaped leaf
*has_location*	Mediterranean
*produces*	acorn

Depending on the kind of semantic information activated in the definition, different images are selected. Holm oak is a physical object and thus preferably represented by color photographs for a high degree of referential similarity. To represent the concept as completely as possible, each proposition of its definitions must be represented. For instance, the following image is a good example of the type_of relation (https://es.wikipedia.org/wiki/Quercus_ilex#/media/Archivo:Mendaza_Navarra_Spanien-Steineiche.jpg). To represent the relations has part and produces, the following image (https://es.wikipedia.org/wiki/Quercus_ilex#/media/Archivo:Bellotas_de_encina_2.jpg) is illustrative. *Has_location* cannot easily be represented by a photograph as the geographical location includes several broad regions. The best option in this case is a map (https://en.wikipedia.org/wiki/Quercus_rotundifolia), which is an abstract drawing that uses color-coding to differentiate between the location of the two subspecies of holm oak, Quercus ilex (pink) and Quercus ilex rotundifolia (green).

**Figure 3:**
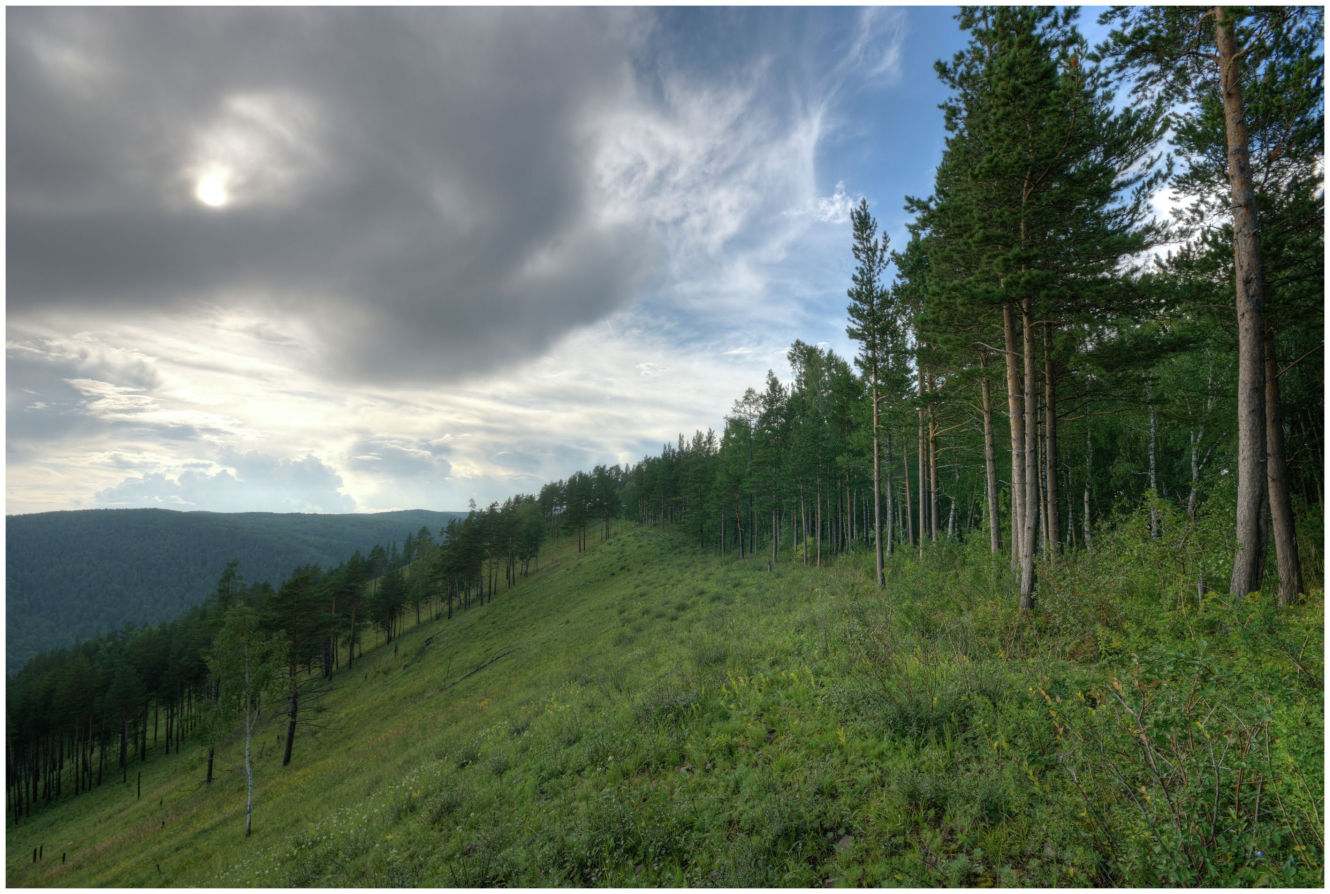
Reproduced with permission. Image licensed under the Creative Commons Public Domain CC0 1.0 Universal, available at https://es.m.wikipedia.org/wiki/Archivo:Krasnoyarsk_Taiga.jpg.

**Figure 4:**
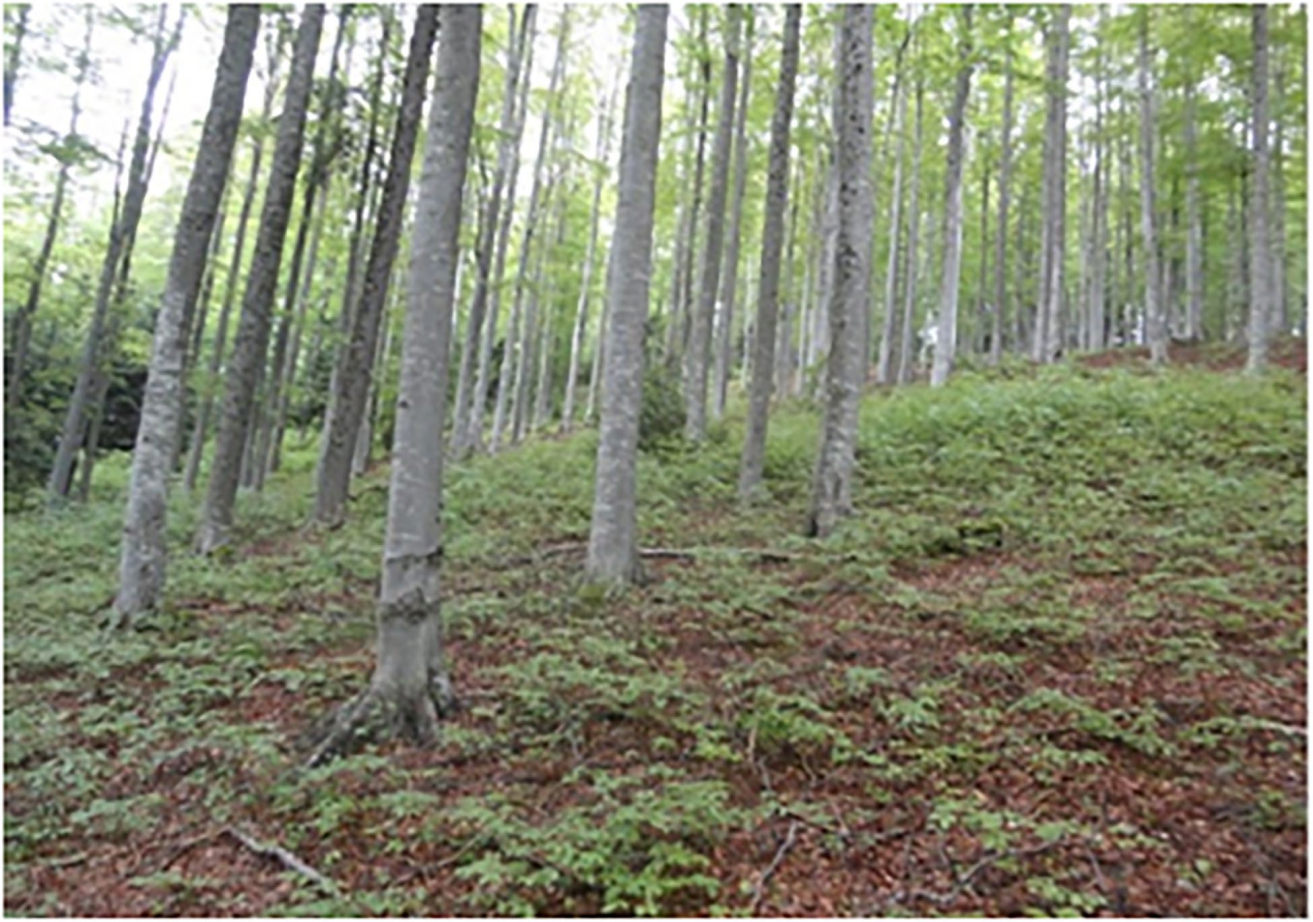
Image for the category of mountainous beech forest. *Source*: LIFE SySTEMiC project. Reproduced with permission from LIFE SySTEMiC project, available at https://www.lifesystemic.eu/.

The location of holm oak and the fact that this tree is the most abundant type of trees there will probably affect the concept that people who were born in the Mediterranean have of tree. Their concept of tree will probably be very similar to a holm oak, whereas the concept that people living in Northern America have of oak is probably more similar to the white oak (*Quercus alba*), which is much more abundant there. Location therefore seems an important starting point for the introduction of cultural aspects in TKBs. The remainder of this paper will analyze and discuss how culture fits in the selection of images for knowledge representation in TKBs.

## Materials and Methods

This exploratory study on the selection of images for cultural aspects of environmental concepts explores two types of contexts: (i) culture and (ii) semantic relations and frames for depicting cultural components (following typology of contexts of Faber and León Araúz, 2016). Therefore, the first step was to develop the definitional template for forest by means of its most prototypical relations and attributes. In FBT, factorization of definitions in existing terminological resources is used as an initial step in definitional template development based on stepwise lexical decomposition of Dik (1978) as applied in Faber and Mairal (1999). The specification and structure of specialized meaning definitions provide key information for establishing semantic networks of specialized concepts. They can serve as the basis of a semantic hierarchy since in definitions, the genus designates the superordinate concept of the defined word, and the differentiating features are the properties that make the concept different from other members of the same conceptual category. Definitions can thus be regarded as mini-knowledge representations that reflect the existence of a definitional frame or template typical of each category (Faber and Medina-Rull, 2017, p. 426).

The *Compilation of Forestry Terms and Definitions* from the European Forest Institute Internal Report No. 62002 (Schuck et al., 2002) was used for this process since our study analyzes forests in a European context and it is the most recent term compilation on the topic. The report compiles 118 forestry terms and several definitions for each from 58 different reliable sources such as the Food and Agriculture Organization of the United Nations and the International Union of Forestry Research Organizations. The definitions of all terms (29) that included the lemma *forest* were analyzed to reconstruct the concept of forest, the conceptual propositions that it displays, and the dimensions that are highlighted in each subtype mentioned.

For example, the report compiles five definitions for forest; one of them is a general definition, while four are definitions by international institutions (Table 4). The report also includes an appendix with the official definitions for forest for each European country. The existence of this appendix alone already shows how different nations and cultures define the concept differently.

**Table 4:** forest definitions in Schuck et al. (2002, p. 15).

forest		
General definition	Complex ecological system in which trees are the dominant life form.	
International definitions	1	Food and Agriculture Organization of United Nations (FAO) definition (2000): **Land** with **tree crown cover** (or equivalent stocking level) of **more than 10 percent** and **area of more than 0.5 ha**. The trees should be able to reach a **minimum height of 5 m at maturity** *in situ*. May consist either of **closed forest** formations where trees of various stories and undergrowth cover a high proportion of the ground; or of **open forest** formations with a continuous vegetation cover in which tree crown cover exceeds 10 percent. **Young natural stands** and all plantations established for forestry purposes which have yet to reach a crown density of 10 percent or tree height of 5 m are included under forest, as are areas normally forming part of the forest area which are temporarily unstocked as a result of **human intervention** or natural causes but which are expected to revert to forest. Includes: Forest nurseries and seed orchards that constitute an integral part of the forest; forest roads, cleared tracts, firebreaks and other small open areas within the forest; forest in national parks, nature reserves and other protected areas such as those of special environmental, scientific, historical, cultural or spiritual interest; windbreaks and shelterbelts of trees with an area of more than 0.5 ha and a width of more than 20 m. Rubberwood plantations and cork oak stands are included.
	2	Food and Agriculture Organization of United Nations (FAO) definition (1990, for Developed countries): **Land with tree crown cover** (stand density) of **more than about 20%** of the area. Continuous forest with trees usually growing more than about **7 m in height** and able **to produce wood**. This includes both **closed forest** formulations where trees of various stories and undergrowth cover a high proportion of the ground and **open forest** formulations with a continuous grass layer in which tree synusia cover at least 10% of the ground.
	3	Food and Agriculture Organization of United Nations (FAO) definition (1990, for Developing countries): **Ecosystem** with a minimum of **10 percent crown cover** of trees and/or bamboos, generally associated with wild flora, fauna and natural soil conditions, and not subject to agricultural practices. The term forest is further divided, according to its origin, into two categories: **natural forest** and **plantation forest**.
	4	**4.1 International Union of Forestry Research Organizations (IUFRO) definition:** **A land area** with a minimum **10% tree crown coverage** (or equivalent stocking level), or formerly having such tree cover and that is being **naturally or artificially regenerated** or that is being afforested.

In Table 4 (bold used for highlighting), the general definition and international definition 3 use variants of *ecosystem* for the genus. The other three definitions use the genus *land* and the specification *tree crown cover* at a certain percentage. The differences in these percentages may point to a different experience of forest in different locations or cultures. Definitions 1 and 2 also specify the minimum height of trees at maturity and make reference to the classification of *open* versus *closed forest*, which refers to its canopy (i.e., the branches and leaves that spread out at the top of a forest forming a type of roof). Definitions 1, 3, and 4 indicate the importance of human intervention (*human intervention* in 1; *natural and plantation forest* in 3; and *naturally or artificially regenerated* in 4). The conceptual propositions to be deduced from these data for the definitional template are as: forest *type_of* ecosystem/land, forest *has_part* tree cover, canopy *attribute_of* forest, origin *attribute_of* forest, size *attribute_of* forest, and height *attribute_of* forest.

The three definitions for natural forest (Table 5) use the genus *forest* and definitions 2 and 3 indicate that they are composed of indigenous trees. Definition 2 then shows classification criteria for natural forests already seen in the definitions for forest: closed/open and degree of human intervention. However, one new dimension comes up: type of trees (*species composition*).

**Table 5:** natural forest definitions in Schuck et al. (2002, p. 21).

natural forest	
1	A **forest** which has evolved as a sequence of **natural** succession but still showing anthropogenic influences. Also, forests that have developed from unmanaged pastures or from fallow land.
2	Natural **forests** are composed of **indigenous trees**, not planted by man. Or in other words forests excluding plantations. Natural forests are further classified using the following criteria: forest formation (or type): **closed/open** degree of **human** disturbance or modification **species composition**
3	A **subset** of forests composed of tree species known to be **indigenous** to the area.

Finally, the definitions for boreal forest (Table 6) highlight the dimensions *location* (*northern regions*; *subpolar region*) and *type of trees* (*conifers*, *coniferous forest*). Definition 2 describes it as an open forest as well.

**Table 6:** boreal forest definitions in Schuck et al. (2002, p. 9).

boreal forest	
1	One of three main forest zones in the world; it is located in northern regions and is characterized by the predominance of conifers.
2	Open coniferous forest growing on swampy ground that is commonly covered with lichen. It is the characteristic vegetation of the subpolar region spanning northern Eurasia, between the colder tundra zone to the north and the warmer temperate zone to the south.

As shown with the example definitions above, important information can be drawn on the dimensions implied in the concept forest: size (land coverage) and height (average height of the trees), canopy (open/closed), type of trees, location, and degree of human intervention (natural forest/plantation forest). The number of dimensions involved shows that the concept is highly multidimensional. Multidimensionality (Rogers, 2004; León Araúz et al., 2013) refers to the way context affects a concept’s behavior and changes conceptual relations, depending on which dimensions of the concept are highlighted. Therefore, the inclusion of these dimensions in the definitional template depends on if we are trying to define forest in general terms (dimensions that apply to all forests in the world) or certain subtypes and the end-users of the TKB. After the factorization of all the definitions in the *Compilation of Forestry Terms and Definitions*, a definitional template^3^ was created (see Section “From Template to Semplate”) for the concept forest that is applicable to all forests.

Before moving on to the question on how to include cultural aspects in the template, an operational definition for culture must be created, as the general definition mentioned in Section 2 is too broad to apply directly. Although we are aware that our initial proposal for this exploratory study is a reduction of everything culture implies, we base our operational definition on grounded cognition of Barsalou (2003), the pragmatic notion of salience (Giora, 1997), and notion of prototypicality of Rosch (1978). The argumentation is that our perceptions are driven by our personal experience and stored in the form of mental representations (grounded cognition). The information most salient for us is therefore the information which is most known or related to us. What is best known to us is what we consider to be prototypical of a concept and is therefore stored as the most characteristic member of a category. Therefore, geographical *location* is a basic dimension in the mental representation of the concept forest and we will take it as the starting point of our study.

Although mental representations are surely influenced by the highly globalized world we live in, *location* is also an important factor to take into account if our aim is to avoid prioritizing one culture over another. Although a person living in central Spain might have a mental representation of the Alps as a prototypical mountain range, we believe they will also have a prototypical mental representation of their local mountain range, which should thus be represented in a TKB on the topic.

By including the cultural dimension *location* in the definitional template, we develop a so-called *semplate* (Section “From Template to Semplate”). Burenhult and Levinson (2008, p. 144) propose the term *semplate* for a template that includes cultural aspects which create, classify, and contrast different categories. We used the EEA Technical Report No 9/2006, *European forest types: Categories and types for sustainable forest management reporting and policy* (European Environment Agency, 2007), to find the most prototypical forest type for each European region and country. Finally, we selected images for each category and annotated them for European region and countries for inclusion in EcoLexicon (Section “Selection of Culturally Adapted Images”). Selection was based on the FBT method for image selection explained in Section “Multimodal Knowledge Representation in Frame-Based Terminology.”

## Results

### From Template to Semplate

By means of the definition factorization explained in Section “Materials and Methods”, we developed the definitional template for forest (Table 7). It was based on a common core of conceptual relations, which can be applied to all types of forests.

**Table 7:** forest definitional template.

forest	
*type_of*	land
*has_part*	tree
*has_attribute*	size
*has_attribute*	canopy
*has_attribute*	origin
*has_attribute*	height

As can be observed, forests can be classified in terms of different dimensions, namely, the type of trees, size, their canopies, origin, tree height, as well as their location, which is an inherent property of the genus, i.e., land. Since definitions must be adapted to end users, some of the relations in this template may not be included in a definition of forest. For example, a forest can be defined as a large area of land covered with trees. In other words, attributes such as canopy, origin, or height can be dispensable in a basic forest definition but could however be included in more specialized descriptions. Similarly, while a particular image of forest would probably not include all these dimensions, the selection of several images would facilitate representation of these different facets. Furthermore, these dimensions give rise to different forest types when a particular dimension is emphasized. For instance, a natural forest specifies the *origin* attribute, while an open or closed forest focuses on a type of *canopy*. As a result, relations can activate specific values in forest hyponyms.

More importantly, many of these parameters are derived from cultural perceptions, especially when the forest is typical of a certain geographic area or region. In particular, the type of trees and its location are dimensions related to culture since climate determines the type of trees that grow in a particular area. Therefore, different types of forests can be found in different locations. For example, as mentioned in Section “Multimodal Knowledge Representation in Frame-Based Terminology,” the holm oak forest is typical of Mediterranean areas. The type of trees thus shows different cultural associations of the forest concept. These culturally determined relations led us to develop the forest semplate, which facilitates inclusion of cultural aspects in the definitional template. This semplate was then particularly useful for the selection of images that represent the culture-specific values of these relations.

As an introduction to the selection of culturally adapted images, we focused on the *location* relation, which explicitly indicates the cultural marker. On the contrary, analyzing the relevance of tree type in culture would entail ascertaining the types of trees typical of different European areas, which would be considerably difficult since often several types of trees are prototypical in a particular region.

The EEA Technical report (European Environment Agency, 2007) identifies 14 European forest types (Table 8) and then indicates where these forest types can be found in Europe. To determine the type of forest that is most prototypical in every European country, we selected the one that had the highest percentage of presence. Thus, following the concepts of salience (Giora, 1997) and grounded cognition (Barsalou, 2003), it was assumed that the mental image of forest for a group of people will be that of the most frequent forest in their particular area. For example, three forest categories can be found in Moldova: (i) mesophytic deciduous forest (80%), (ii) thermophilous deciduous forest (10%), and (iii) plantations and self-sown exotic forest (10%). Since the first one is the most frequent, it was selected as the prototypical forest in Moldova. Table 8 shows the 14 categories of European forest types described in the EEA Technical report (European Environment Agency, 2007), together with the countries where every forest type is the most prototypical.

**Table 8:** EEA Technical report (European Environment Agency, 2007) forest types and countries where they are prototypical.

1 Boreal forest: Finland Norway Sweden 2 Hemiboreal forest and nemoral coniferous and mixed broadleaved coniferous forest: Belarus Czech Republic Estonia Germany Latvia Lithuania Luxembourg^1^ Poland Russia 3 Alpine coniferous forest: Andorra Austria Bulgaria Slovak Republic Switzerland 4 Acidophilous oak and oak-birch forest: Luxembourg 5 Mesophytic deciduous forest: France Luxembourg Moldova 6 Beech forest: Croatia Luxembourg Romania 7 Mountainous beech forest: Slovenia 8 Thermophilous deciduous forest: Italy Serbia 9 Broadleaved evergreen forest: Azores Portugal 10 Coniferous forest of the Mediterranean, Anatolian and Macaronesian regions: Canaries Cyprus Greece Spain 11 Mire and swamp forests: 12 Floodplain forest: 13 Non riverine alder, birch or aspen forest: 14 Plantations and self-sown exotic forest: Belgium Czech Republic Hungary Ireland Netherlands United Kingdom

1 *Four forest types (2, 4, 5, and 6) had exactly the same frequency in Luxembourg. Therefore, all four types were included as prototypical in this country.*

Table 8 shows that 11 of these forest types were prototypical in at least one European country. The remaining three types also exist in Europe but were not prototypical in any country. The semplate of these forest types was filled by completing the forest template with specific values and adding cultural markers, such as the *location* relation. This was used to select and annotate different images of a concept based on cultural perceptions. As an example, Table 9 describes the alpine coniferous forest semplate.

**Table 9:** alpine coniferous forest semplate.

alpine coniferous forest	
*type_of*	forest
*has_part*	coniferous trees, mainly *Picea abies*, *Abies alba*, *Pinus sylvestris* and *Pinus mugo*
*has_location*	Andorra, Austria, Bulgaria, Slovak Republic, Switzerland

Even though the EEA Technical report (European Environment Agency, 2007) also alludes to their presence in other types of geographical entities (i.e., the Pyrenees, the Alps, the Apennines, the Carpathians, and the Scandinavian Alps), cultural labels in this exploratory study were European countries, because these facilitate the selection of culturally adapted images and avoid overlap.

Semplates show the relations that acquire specific values in the concept in question. That is to say, some relations of the template do not appear in the semplate because their values are not specified in the concept but rather in their hyponyms. This is the case of origin, size, canopy, and height. For example, an alpine coniferous forest can be either an open forest or a closed forest, depending on the type of alpine coniferous forest in question. Therefore, for the sake of clarification and comparison with other concepts, just specific characteristics of the concept were described in the semplate.

### Selection of Culturally Adapted Images

**Figure 5:**
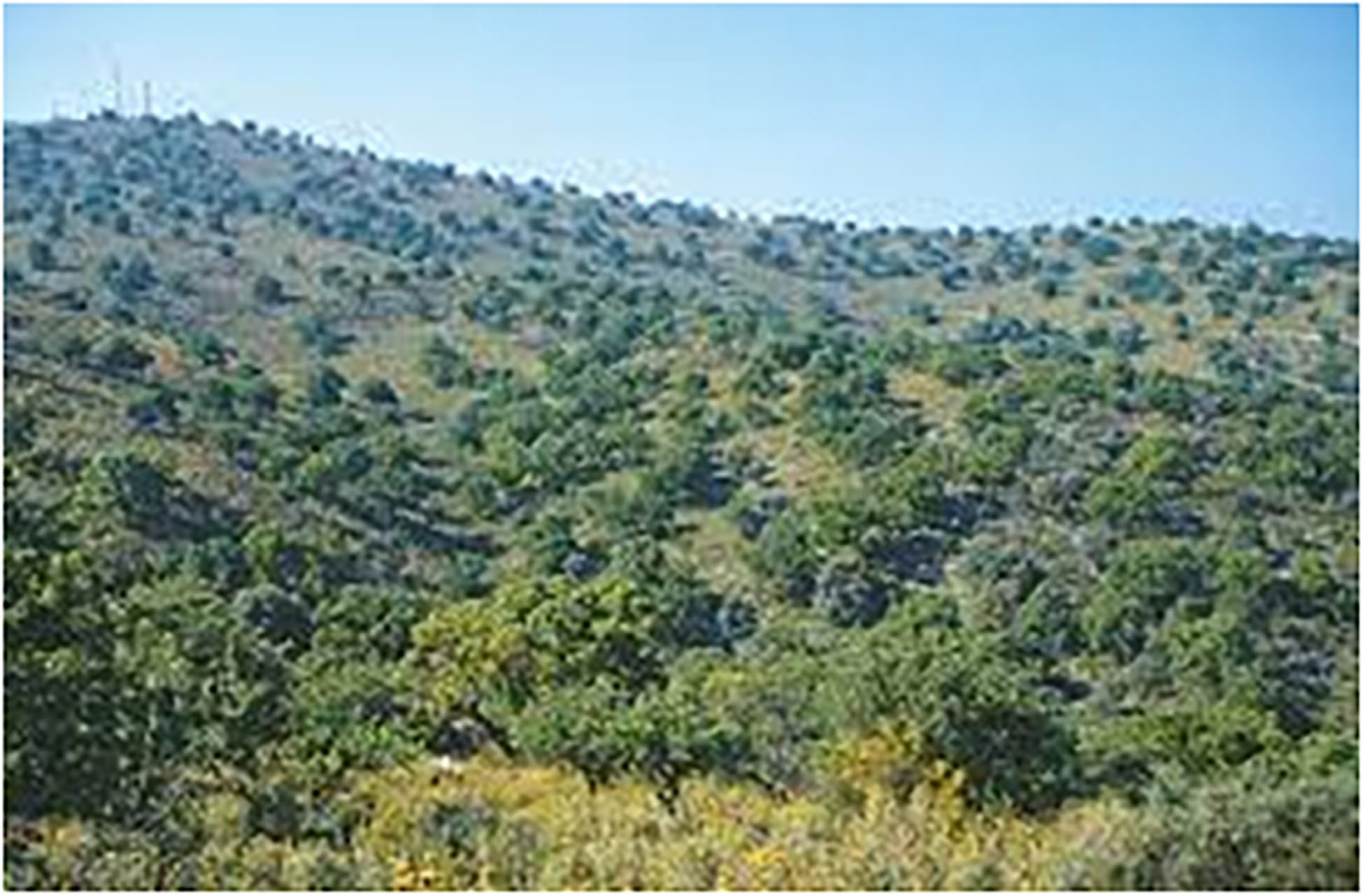
Image for the category of thermophilous deciduous forest. *Source*: European Red List of Habitats—Forests Habitat Group. Reproduced with permission from Stamatis Zogaris, available at https://acortar.link/dM3PWV.

**Figure 6:**
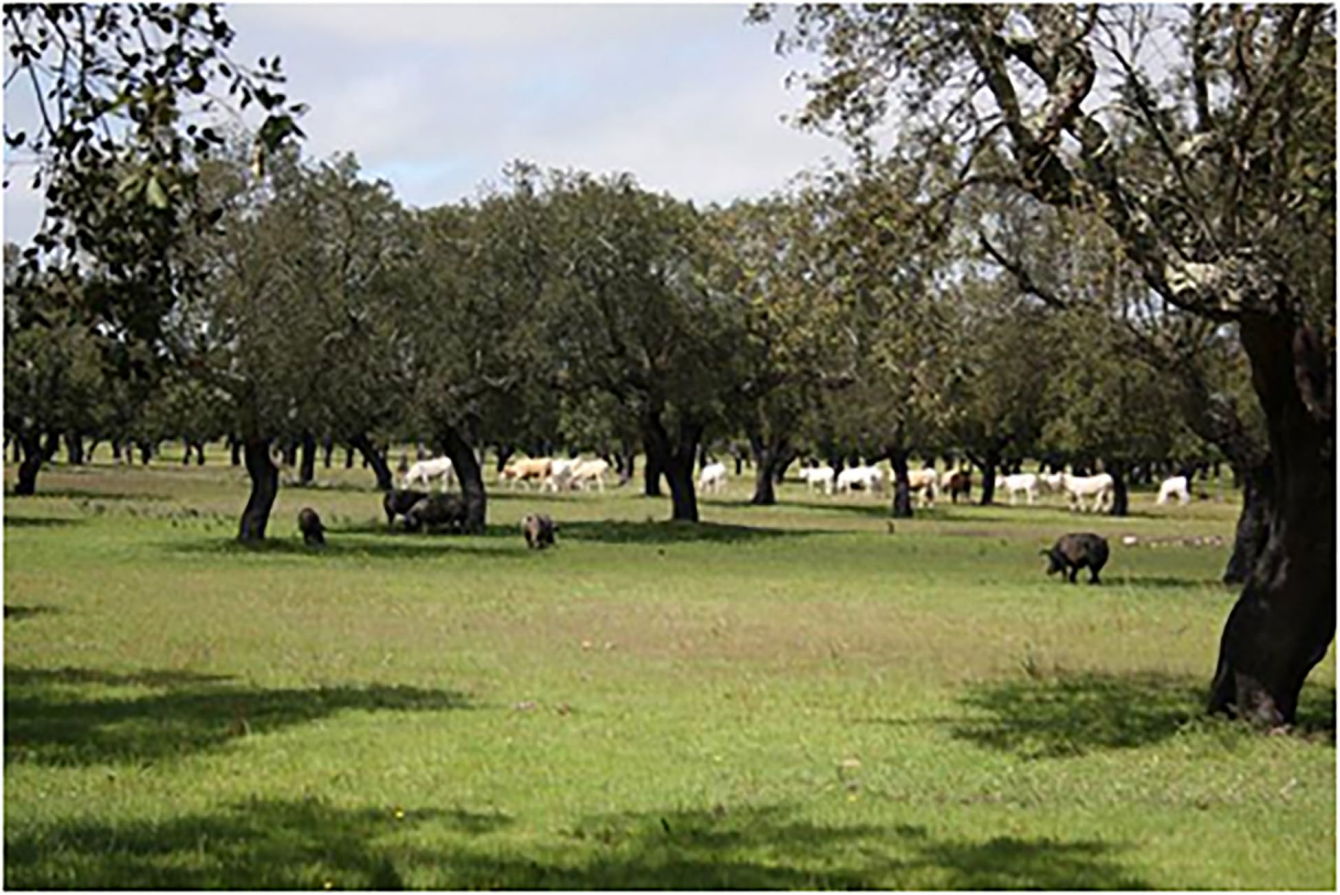
Image for the category of broadleaved evergreen forest (Portugal). *Source*: AGFORWARD project. Reproduced with permission from João HN Palma, AGFORWARD project, available at https://www.agforward.eu/montado-in-portugal.html.

Different types of forests are prototypical in European countries. Therefore, if just one image is included for forest in a TKB, cultural diversity is not acknowledged, and this could mean that specific cultural ways of thinking about forests are imposed upon other cultures. To account for the different ideas of forest in Europe, this pilot study describes the selection of an image for every forest type that was found to be prototypical in a European country. Since salience is a subjective concept that guides language production (Myachykov, 2007) and processing (Giora, 1997), it would help to include several images, because concepts are culturally malleable. The following images (Figures 3–6) were chosen according to the principle of referential similarity (Section “Multimodal Knowledge Representation in Frame-Based Terminology”), which must be met when selecting images in EcoLexicon,^4^ the TKB that will include the results of this study. As the different forest types are geographical entities, color photographs which provide the highest degree of referential similarity, are the most adequate for inclusion in a TKB.

As can be seen in Figures 3–6, the inclusion of several images makes it possible to account for the different cultural conceptions of forest. For example, a forest for Norwegian people (Figure 3) is radically different from a forest for Portuguese people (Figure 6). Furthermore, annotating the images with the country where every forest type is most salient can be used to obtain a dataset of images tagged with one or more countries, which is very useful to carry out advanced queries in the TKB.

Nevertheless, even though referential similarity is a fundamental requirement in image selection, it would be redundant to find several images that are quite similar. For example, Figure 4 shows, a beech forest and a mountainous beech forest. Unfortunately, this dimension is not particularly evident in the image since it is difficult to find an image that clearly illustrates both the type of trees and its mountainous location. However, thanks to this, Figure 4 can also be used to represent a beech forest, thus avoiding image overload.

Other images found for some of the categories (mesophytic deciduous forest and acidophilous oak and oak-birch forest) did not show the size dimension of forest, as they zoom in on a few trees, thus be more adequate to represent the specific hyponym of forest and the type of trees in those forests.

Therefore, in an effort to develop a culture inclusive TKB by means of adapted images, terminographers would have to decide whether 11 images are necessary in the forest entry or whether they would rather cause an overload of redundant information. This study argues that images that are different from each other should ideally be prioritized with a view to avoiding information overload. Evidently, even though not all images will be included in the forest entry, they would be present in each hyponym’s entry. Moreover, to avoid information overload, we propose that images of forest types that are prototypical in multiple countries should be prioritized in order to reach a larger audience. For example, alpine coniferous forests are typical in more countries than boreal forests (Figure 3). The forest types in the EEA Technical report (European Environment Agency, 2007) were created because of the need to have a classification at the European level, in addition to the multiple classifications already existing in every country. However, this unifying effort resulted in categories that sometimes turn out to be administrative, i.e., they have been created for this specific purpose, and therefore include very different subtypes. This is the case of category 9, broadleaved evergreen forest, which is prototypical in the Azores and Portugal. Choosing an image of broadleaved evergreen forest that is representative of these two countries is extremely difficult, because a different subtype of this category prevails in each of these countries. These are influenced, among other aspects, by their different climates. Thus, our proposal is to include two images, which describe two subtypes of broadleaved evergreen forest: (i) an image showing a Macaronesian laurisilva, which is the prototypical forest type in Azores, and (ii) an image showing a Mediterranean evergreen oak forest (also known as montado), which is the most salient forest type in Portugal (Figure 6).

The use of coarse-grained categories also complicates their representation in TKBs, because some categories do not respond to “real” forest types. An example can be found in category 2, hemiboreal forest and nemoral coniferous and mixed broadleaved-coniferous forest. Not only does this designation include three forest types, but the EEA Technical Report (European Environment Agency, 2007) specifies six subtypes of this category, which are prototypical of different European regions. However, the assignment of a particular subtype to a specific country is not provided in the document (as is the case of Azores and Portugal) and would probably entail rethinking the forest categorization as well as the use of countries or additional geographical tags, such as regions or climate areas. Therefore, different images for every culture could not be selected in this case. Not surprisingly, forest categorization, as well as the selection of prototypical images, are not easy tasks. Based on the results from this exploratory study, it would be interesting to develop a finer-grained categorization, which would enhance the selection of images and the representation of culturally bound concepts in terminological resources.

However, the analysis described in this section also highlights the benefits of this approach. One of them is the greater representativeness, as a more complete description of the concept is provided, which would be difficult by means of a single image. For example, the images selected show parameters that vary in different forest types, such as tree type, open canopies (Figures 5, 6), and closed canopies (e.g., Figure 3), among other. All of them provide a full picture of the forest concept in different cultures. In addition, such a culturally inclusive TKB would facilitate knowledge acquisition to users from different cultures since they will find images adapted to their culture. In summary, this improves usability of the resource and user satisfaction.

Moreover, images can be reused to describe additional concepts. Figure 5 is a very nice representation of an open forest; it can therefore be used for the concept entry forest to highlight the canopy dimension, for the thermophilous deciduous forest entry and for the open forest entry.

Furthermore, as a result of multidimensionality, these images also account for other conceptual dimensions of forests and thus can be reused to describe terms and concepts that make these dimensions explicit. For example, the image of boreal forest (Figure 3) could also be used in the coniferous and coniferous forest entries, because this type of trees category (and the resulting forest) is clearly described in the image. Furthermore, the evergreen and evergreen forest entries could also include this image, which clearly shows this type of leaf, as well as the closed canopy entry, because the image represents a dense growth of trees in which the top branches and leaves form a sort of ceiling. The natural forest and high forest entries could also benefit from this image, which represents additional dimensions described in the forest template and semplate.

## Conclusion

As culture is underrepresented in terminological resources, in this paper, we present an exploratory study on how to select images for culturally bound concepts in terminological knowledge bases. Although the study focuses only on the concept forest in a European context, the methodology can be applied to other continents and regions in the world.

Frame-Based Terminology provided the theoretical premises and methodological background for multimodal knowledge selection and representation. Furthermore, following the premises of FBT, the definitional template of forest was converted into a definitional semplate, where the *location* and *tree type* dimensions were found to be the most related to culture. This study focused on the *location* dimension and, with the help of this semplate, images were selected to represent the prototypical forest for each European country. The results of this study showed that images of forests that are salient in cold regions (e.g., Figure 3) are completely different from forests typical of warmer regions (e.g., Figure 6). Therefore, the inclusion of several images is an excellent option to account for the different ideas of forest in Europe.

However, this proposal is not without limitations. First, we adopted a European perspective, which evidently provides a partial view of the multiple cultural perceptions of forest. Furthermore, the granularity of the categorization used largely determines the output of this proposal, as described in Section 5. Another limitation to this study, although applicable to all terminographical work, is the need for continuous updating of the images in the TKB. Forests change over time due to many factors. One of them is human interference: direct intervention, in cases of reforestation and afforestation, and indirect interference when, for example, climate change causes the Mediterranean beech forest to invade more northern regions. Therefore, we believe that future research should address the dimensions of time and human intervention and their relation to culture. Plans for future research also include a survey conducted among people of different European countries to ascertain their perceptions of the most prototypical type of forest in different European areas. Furthermore, the role of languages in the selection of culture-bound prototypical images will also be addressed.

Another line of research involves the selection of images for forests in other continents and the possibility of joining countries into bigger areas when they present the same forest type and thus a similar cultural representation of forest. This may avoid overburdening the TKB in the same way as the reuse of images does when annotating other dimensions (see Section “Selection of Culturally Adapted Images”).

## Data Availability Statement

The original contributions presented in the study are included in the article/supplementary material, further inquiries can be directed to the corresponding author.

## Author Contributions

MC-G and AR conceived and designed the study and contributed to manuscript writing and revision. All authors contributed to the article and approved the submitted version.

## Funding

This research was carried out as part of project PID2020-118369GB-I00, Transversal integration of culture into an environmental terminological knowledge base (TRANSCULTURE), funded by the Spanish Ministry of Science and Innovation and project A-HUM-600-UGR20, La cultura como módulo transversal en una base de conocimiento terminológico medioambiental (CULTURAMA), funded by the European Regional Development Fund (FEDER).

## Conflict of Interest

The authors declare that the research was conducted in the absence of any commercial or financial relationships that could be construed as a potential conflict of interest.

## Publisher’s Note

All claims expressed in this article are solely those of the authors and do not necessarily represent those of their affiliated organizations, or those of the publisher, the editors and the reviewers. Any product that may be evaluated in this article, or claim that may be made by its manufacturer, is not guaranteed or endorsed by the publisher.

## Footnotes

^1^ https://ecolexicon.ugr.es/

^2^The definitions for these concepts have not yet been implemented in EcoLexicon.

^3^The definition of forest has not yet been implemented in EcoLexicon.

^4^To include images in a TKB, copyright issues should also be considered. In this paper, we explain the methodology applied to select images, but copyright for the images shown has not been taken into account. Nevertheless, images under a creative commons license or certain images from Wikipedia and those included in publications by the US Federal Government can be freely reproduced, for example.
